# Nafion‐Induced Reduction of Manganese and its Impact on the Electrocatalytic Properties of a Highly Active MnFeNi Oxide for Bifunctional Oxygen Conversion**

**DOI:** 10.1002/celc.202100744

**Published:** 2021-08-16

**Authors:** Dulce M. Morales, Javier Villalobos, Mariya A. Kazakova, Jie Xiao, Marcel Risch

**Affiliations:** ^1^ Nachwuchsgruppe Gestaltung des Sauerstoffentwicklungsmechanismus Helmholtz-Zentrum Berlin für Materialien und Energie GmbH Hahn-Meitner-Platz 1 14109 Berlin Germany; ^2^ Boreskov Institute of Catalysis SB RAS Lavrentieva 5 630090 Novosibirsk Russia; ^3^ Department of Highly Sensitive X-ray Spectroscopy Helmholtz-Zentrum Berlin für Materialien und Energie GmbH Albert-Einstein-Straße 15 12489 Berlin Germany

**Keywords:** bifunctional oxygen electrodes, Nafion, oxidation state, transition metals, X-ray absorption spectroscopy

## Abstract

Electrocatalysts for bifunctional oxygen reduction (ORR) and oxygen evolution reactions (OER) are commonly studied under hydrodynamic conditions, rendering the use of binders necessary to ensure the mechanical stability of the electrode films. The presence of a binder, however, may influence the properties of the materials under examination to an unknown extent. Herein, we investigate the impact of Nafion on a highly active ORR/OER catalyst consisting of MnFeNi oxide nanoparticles supported on multi‐walled carbon nanotubes. Electrochemical studies revealed that, in addition to enhancing the mechanical stability and particle connectivity, Nafion poses a major impact on the ORR selectivity, which correlates with a decrease in the valence state of Mn according to X‐ray absorption spectroscopy. These findings call for awareness regarding the use of electrode additives, since in some cases the extent of their impact on the properties of electrode films cannot be regarded as negligible.

## Graphical abstract

**Binder's fee**: The impact of Nafion on the properties of MnFeNi oxide nanoparticles as a highly active, bifunctional oxygen reduction/oxygen evolution reaction (ORR/OER) catalyst is investigated by voltammetric methods and X‐ray absorption spectroscopy. The ORR/OER activity of the catalyst increases in the presence of Nafion due to an improved particle contact, in addition to a binder‐induced Mn reduction that results in highly selective ORR sites towards the direct conversion of O_2_ to OH^−^.

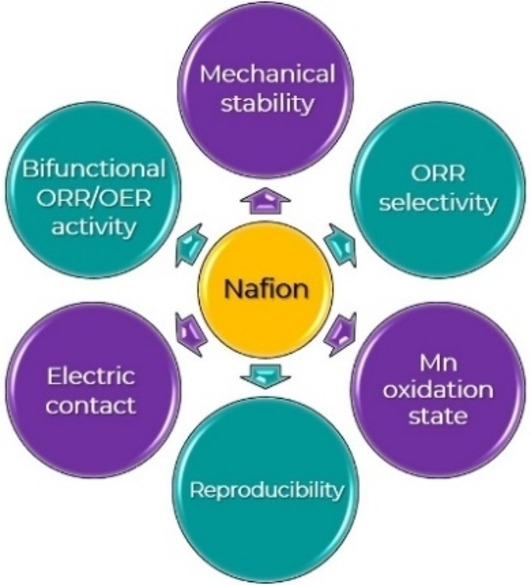

Electrocatalytic oxygen conversion–comprising the oxygen evolution (OER) and the oxygen reduction reaction (ORR)–is considered a major obstacle for the commercialization of green, regenerative energy conversion technologies, for example, reversible fuel cells or rechargeable metal‐air batteries, since both reactions suffer from sluggish kinetics.^[1]^ Hence, the design of low‐cost, high‐performance bifunctional ORR/OER electrocatalysts (BOEs) is essential to increase the efficiency of these devices.^[2]^ Investigation of BOEs is typically conducted under hydrodynamic conditions using rotating disk electrode (RDE) setups or electrochemical flow cells.[^3^, ^4^, ^5^] It is thus necessary that the mechanical stability of the investigated electrode film is sufficiently high to endure the experimental conditions required for the evaluation of its catalytic properties. This can be achieved by means of a binder, which not only aids in preventing catalyst detachment, but also in improving the electric contact between catalyst particles and electrode substrate, lowering thus the resistance of the electrode film.[^6^, ^7^] Depending on the binder and its concentration, additional effects on various properties of electrode films have been reported, including ionic conductivity, hydrophobicity, mass transport, and accessibility to the reaction sites, often leading to an improvement of the catalytic performance.[^8^, ^9^, ^10^]

A popular compound used as a binder is Nafion, an ion‐conducting ionomer comprising hydrophilic, sulfonic‐terminated side chains, and built upon copolymerization of tetra‐fluoroethylene and perfluorinated vinyl ether monomers.^[11]^ This binder has proved successful in improving the mechanical stability of electrode films, and thus it has been recommended for benchmark electrode preparation protocols.[^12^, ^13^] It is reported that Nafion may induce decreases in overpotential attributed to an increased electrical conductivity and facilitated mass transport,^[6]^ though depending largely on the electrode composition,[^8^, ^12^, ^14^] and not impacting otherwise the catalytic activity of, for instance, IrO_2_,[^10^, ^12^] Pt/C,[^6^, ^15^] or Pt−Sn/C.^[15]^ However, the nature of additional effects observed with diverse materials remains unclear. It has been speculated that Nafion impacts the intrinsic catalytic properties of Pt, attributed to the specific adsorption of sulfonate groups on the catalyst surface.^[8]^ Moreover, it was recently reported that the OER activity trend exhibited by Mn oxides of various crystal structures was different in the presence of Nafion than in its absence, speculatively due to a binder‐induced chemical change of the surface of these materials.^[16]^ Furthermore, the acidic nature of Nafion may also lead to corrosion and catalyst dissolution in the case of materials that are not chemically stable in low pH media,^[5]^ impacting further the apparent activity of the investigated catalyst films. Hence, understanding the influence of Nafion on the catalytic properties of materials under investigation results crucial for studies that aim to elucidate their intrinsic properties.

As a case study, we investigate multiphase Mn, Fe and Ni oxide nanoparticles (Mn_0.51_Fe_0.14_Ni_0.35_ metal composition and 14.4 wt % total metal loading) supported on oxidized multi‐walled carbon nanotubes, hereafter denoted MnFeNiOx, which was recently proposed as a high‐performance BOE.^[17]^ According to a previous TEM study, the nanoparticles are located inside and outside the walls of the nanotubes in a 1.25 : 1 ratio, with an average particle size of 3.6±1.2 nm and 10.5±5.9 nm.^[17]^ HAADF STEM and EDX elemental analysis (Figure S1) indicates that each particle has its own individual composition. Yet, the composition averaged to 50 nm was Mn_0.51_Fe_0.18_Ni_0.31_, which is in fair agreement with previous XRF analysis.^[17]^ Additionally, the elemental maps shown in Figure S1 suggest that the three metals are likely to be exposed to Nafion during the treatments described in the following. Catalyst inks (MnFeNiOx dispersed in a 1 : 1 water‐ethanol mixture) were deposited onto glassy carbon (GC) RDEs in the presence or absence of 2 vol % Nafion solution (∼5% Nafion in a mixture of alcohols) in order to observe its impact on the electrocatalytic properties of the obtained catalyst films. The binder‐containing and binder‐free films are hereafter denoted MnFeNiOx‐Nafion/GC and MnFeNiOx/GC, respectively. Linear sweep voltammograms (LSVs) of MnFeNiOx/GC and MnFeNiOx‐Nafion/GC were recorded in triplicate in the ORR and OER potential regions. The individual measurements and their averages are shown in Figure S2 and Figure 1a, respectively. To facilitate the comparison, we determined the potential at which disk current densities of +10 mA cm^−2^ (*E_OER_*) and −1 mA cm^−2^ (*E_ORR_*) were attained for the two samples as activity metrics[^4^, ^18^] (Table S1). The obtained *E_OER_* values were 1.547±0.006 and 1.538±0.007 V vs RHE for MnFeNiOx/GC and MnFeNiOx‐Nafion/GC, respectively, indicating only a slight increase in the apparent OER activity of the electrodes upon addition of Nafion, which could be explained by an improved contact between the conductive MnFeNiOx powder and the GC substrate,^[10]^ while differences in mass transport and availability of the active sites may also play a role. Similarly in the case of the ORR, MnFeNiOx‐Nafion/GC displayed an *E_ORR_* value of 0.788±0.003 V vs RHE, while for MnFeNiOx/GC *E_ORR_* was 0.776±0.009 V vs RHE, with a comparatively higher reproducibility in the case of the former as shown in Figure S2. Yet, a more substantial difference in the recorded ORR currents was observed in the kinetic‐diffusion mixed control region, which could be related to differences in ORR selectivity. To investigate this, LSVs were recorded at different electrode rotation rates, and the number of electrons transferred during the ORR (*n*) was determined via the Koutecky‐Levich (K‐L) analysis.[^19^, ^20^] The analysis was conducted from three independent sets of measurements (Figure S3) extracting data at 0.55, 0.60, 0.65 and 0.70 V vs RHE (Figure S4), and displaying high reproducibility in the investigated potential range (Table S2). Average LSVs and K‐L plots obtained at 0.65 V vs RHE are displayed in Figure 1b and 1c, respectively. For MnFeNiOx/GC, *n* had a value of 2.3, which translates into a pathway favoring the formation of peroxide species (*n*=2), according to Equation (1).^[20]^ Interestingly, for the Nafion‐containing sample *n* was 3.8, indicating that the direct reduction of O_2_ to OH^−^ is favored [Eq. (2)],^[20]^ which agrees with an earlier study conducted by rotating ring disk electrode voltammetry.^[17]^ These results indicate that the presence of Nafion leads to a major improvement of the ORR selectivity of the trimetallic catalyst.

(1) $$O_{2}+H_{2}O+2e^{-}\to {HO}_{2}^{-}+{OH}^{-}$$

(2) $$O_{2}+{2H}_{2}O+4e^{-}\to 4{OH}^{-}$$

**Figure 1:**
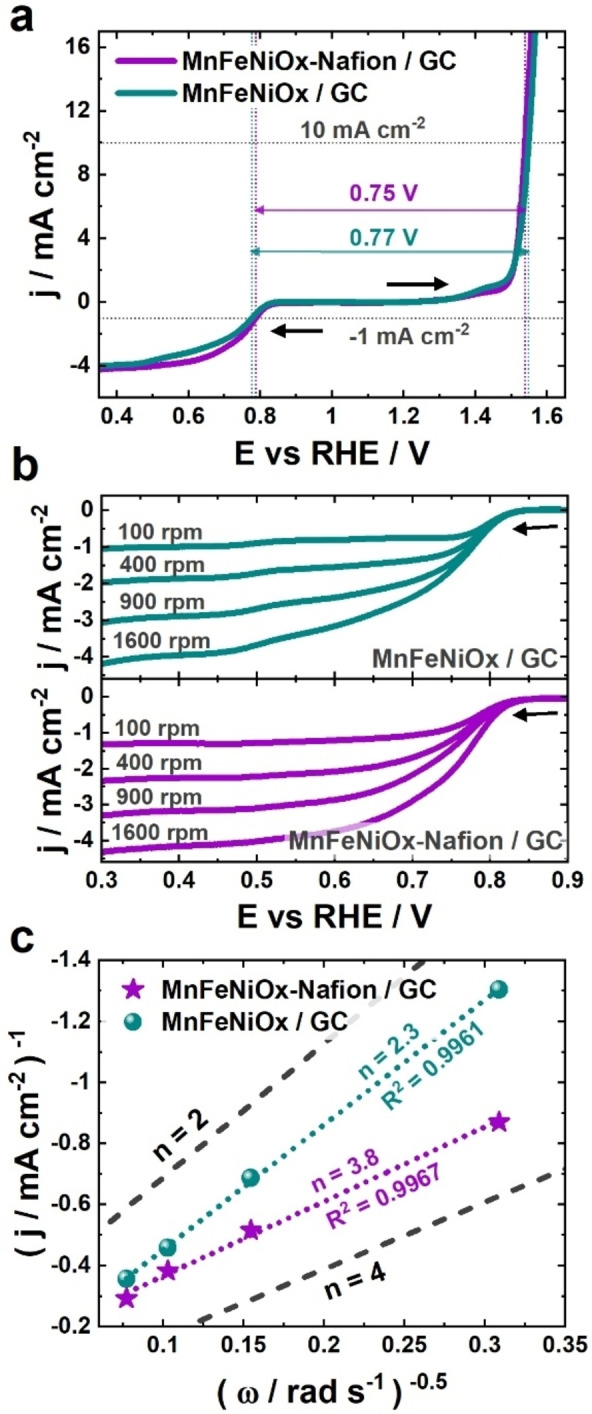
*iR_U_*‐drop‐compensated LSVs corresponding to MnFeNiOx deposited onto GC‐RDEs in the presence (purple) and in the absence (teal) of Nafion with a scan rate of 5 mV s^−1^ (a) at 1600 rpm electrode rotation in the OER and ORR potential regions, (b) at different electrode rotation rates in the ORR potential region, and (c) their corresponding Koutecky‐Levich plots obtained at 0.65 V vs RHE. Simulated plots corresponding to the transfer of 2 and 4 electrons are shown for guidance, and were determined considering *D*=1.9×10^−5^ cm^2^ s^−1^, *ν* =1.1×10^−2^ cm^2^ s^−1^, and *C*=1.2×10^−6^ mol cm^−3^.^[20]^ All measurements were conducted in O_2_‐saturated 0.1 m NaOH solution. Black arrows indicate the direction of the voltammetric scan.

MnFeNiOx was initially proposed as a two‐component catalyst with FeNiOx (3 : 7 metal ratio) being the active site for the OER,^[21]^ and MnOx being the key component that activates the catalyst towards the ORR.^[17]^ If this assumption is correct, and given that the presence of the binder led to a substantial enhancement of the ORR performance while barely influencing the OER activity, it can be hypothesized that the catalyst undergoes Nafion‐induced chemical changes related to Mn. Since correlations between Mn valence and ORR selectivity have been established,[^22^, ^23^] we resorted to X‐ray absorption spectroscopy (XAS) for an in‐depth investigation of our hypothesis.

XAS spectra were collected before (MnFeNiOx) and after (MnFeNiOx(Nafion)) treating the catalyst in a Nafion‐containing water‐ethanol solution by sonication for 15 min (see sample preparation protocol in Supporting Information). The spectra obtained in the Ni‐L_3_, Fe‐L_3_ and Mn‐L_3_ edges are shown in Figure 2a, 2b and 2c, respectively, displaying alongside the spectra of metal oxides of unmixed oxidation states for reference. While no substantial differences in the Ni‐L_3_ spectra were shown by MnFeNiOx and MnFeNiOx(Nafion) (Figure 2a), in the case of the Fe‐L_3_ edge, a change in the background was observed at energies above ∼712 eV (Figure 2b), attributed to the F‐K edge absorption of Nafion's fluorine atoms (Figure 2d).^[24]^ Yet, the prominent Fe‐L_3_ peak did not feature any other visible change. In the case of the Mn‐L_3_ energy region (Figure 2c), the spectra recorded for MnFeNiOx (teal) and MnFeNiOx(Nafion) (purple) displayed major differences. Earlier, XRD characterization revealed that MnFeNiOx consists of various mono and bimetallic oxide phases in a highly defective state.^[17]^ Thus, Mn in MnFeNiOx is expected to be present in a mixture of oxidation states, as observed in Figure 2c, with a larger prevalence of Mn^2+^ and Mn^3+^ surface species according to XPS analyses.^[17]^ The more intense feature centered at ∼641 eV in the spectrum of MnFeNiOx(Nafion) compared to MnFeNiOx suggests that Mn became mainly present in oxidation state 2+ upon exposure to Nafion‐containing solution. Given that Fe and Ni spectra did not display any evident change that correlates with a variation of their oxidation states, it can be assumed that the majority of the binder‐caused surface state changes that led to an improvement of the ORR selectivity are related to Mn. This observation supports the hypothesis that Mn oxide is an essential component of the ORR active sites in MnFeNiOx.

**Figure 2:**
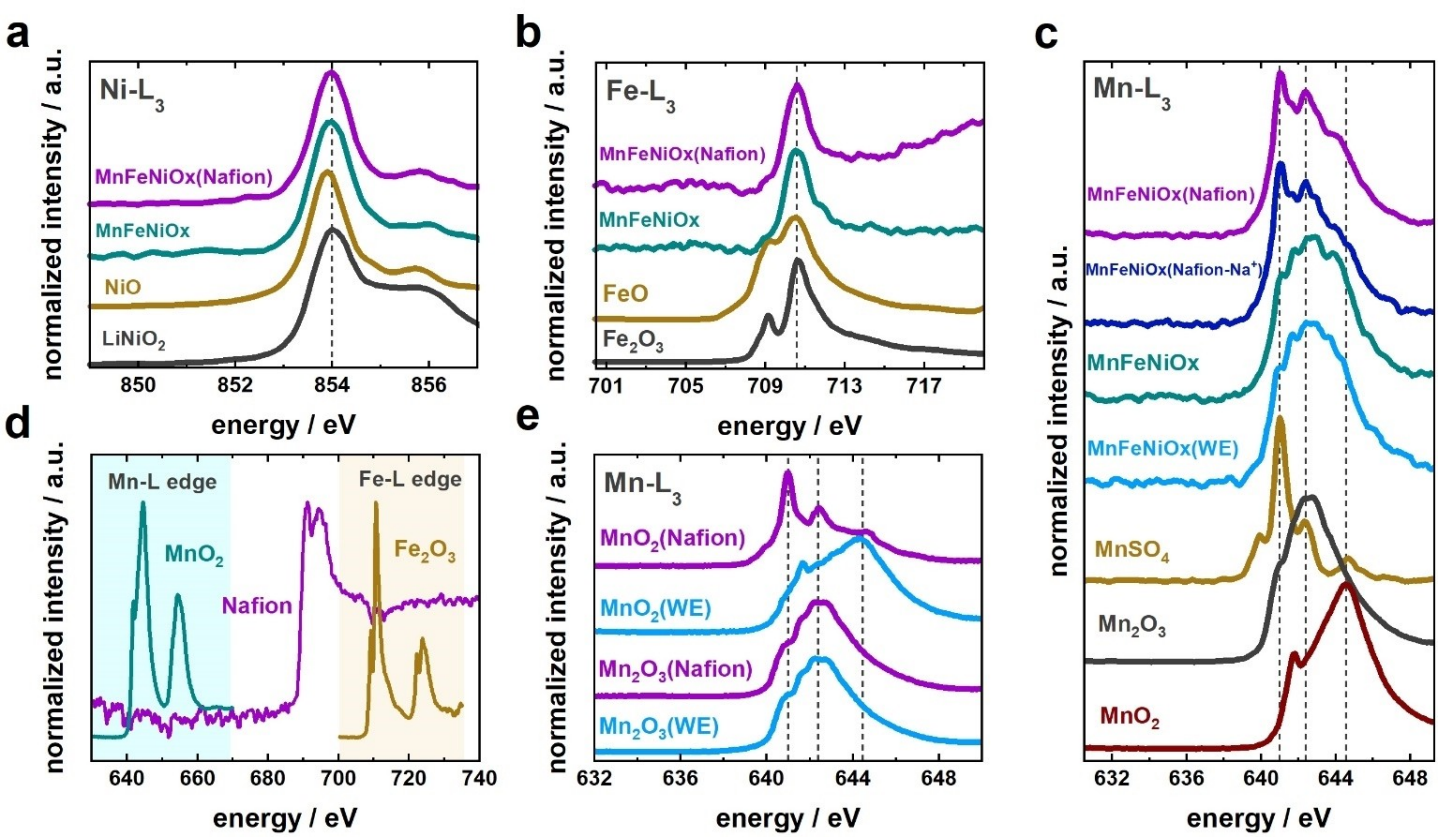
Normalized XAS spectra of MnFeNiOx recorded in total electron yield mode before and after treatment in water‐ethanol mixtures without (WE) or with 2 vol % binder solution (Nafion), recorded in the L_3_ edge of a) Ni, b) Fe, and c) Mn, showing corresponding reference compounds; c) shows additionally the spectra of MnFeNiOx exposed to a WE solution containing cation‐exchanged binder (Nafion(Na^+^)). d) XAS spectrum of Nafion in the energy region comprising both the L_2_‐L_3_ edge of Mn and the L_2_‐L_3_ edge of Fe; the spectra of MnO_2_ and Fe_2_O_3_ are shown for reference. e) XAS spectra of MnO_2_ and Mn_2_O_3_ recorded in the L_3_ edge of Mn after treatment in water‐ethanol mixture in the abscence (WE) and in the presence of binder (Nafion). Spectra were offset for clarity and vertical dashed lines are included to guide the eye.

The increase in the intensity of the spectral feature related to Mn^2+^ species could be attributed to a Nafion‐induced chemical reduction, which has been observed previously with Mn‐containing complexes.^[25]^ However, another plausible explanation is that the strongly acidic proton in the binder induces disproportionation of Mn^3+^, forming Mn^2+^ and Mn^4+^,^[26]^ and resulting in an apparent increase in Mn^2+^ species due to a variation in the ratio Mn^2+^:Mn^3+^:Mn^4+^. To further understand the origin of the change in Mn oxidation state, two control experiments were conducted with MnFeNiOx treated in (1) a water‐ethanol mixture in the absence of Nafion (MnFeNiOx(WE)), and in (2) a solution containing cation‐exchanged Nafion (MnFeNiOx(Nafion‐Na^+^)), namely, after having replaced the H^+^ at the sulfonate groups with Na^+^ according to a previously reported procedure.^[5]^ The quality of the dispersions obtained when using cation‐exchanged Nafion solution did not display any evident difference than that observed with Nafion in its acidic form. The obtained XAS spectra (Figure 2c) show that, while no substantial spectral difference was observed between untreated MnFeNiOx (teal) and MnFeNiOx(WE) (light blue), the changes observed with MnFeNiOx(Nafion) (purple) were displayed as well by MnFeNiOx(Nafion‐Na^+^) (dark blue), thereby suggesting that the acidic proton in the binder does not play a major role in the observed Mn valence changes.

We further investigated the effect of Nafion on the XAS features of MnO_2_ and Mn_2_O_3_, for which the corresponding powders were treated in a water‐ethanol mixture in the absence (Mn_X_O_Y_(WE)) or presence (Mn_X_O_Y_(Nafion)) of the binder (Figure 2e). Interestingly, the peak assigned to Mn^2+^ (641 eV) was clearly seen with MnO_2_ (Mn^4+^) upon exposure to Nafion, whereas the spectra corresponding to Mn_2_O_3_ (Mn^3+^) did not display any substantial change, indicating that the valence changes are not related to disproportionation. We speculate that a strong chemical interaction between Mn^4+^ species in MnO_2_ and the electron donors in the binder takes place. Likely, this occurs similarly with Mn^4+^ species in MnFeNiOx, thus leading to an overall improvement in the ORR performance of the catalyst: on the one hand, the formation of Mn^2+^ species (from Mn^4+^) could favor the binding of *OOH intermediates according to recent DFT predictions,^[27]^ and on the other hand, the unaffected Mn^3+^ atoms provide O_2_ absorption sites^[28]^ and a Mn^3+^:Mn^4+^ ratio that facilitates the 4‐electron transfer pathway.[^23^, ^29^]

In summary, we investigated the influence of Nafion on the electrocatalytic properties of MnFeNiOx as a bifunctional ORR/OER catalyst. Besides the advantageous, yet expected, improvement of electrode film properties (mechanical stability and particle contact), a major benefit on the ORR selectivity of MnFeNiOx was revealed: while binder‐free MnFeNiOx displayed a preferred 2‐electron transfer pathway, in the presence of Nafion the composite electrode exhibited the reduction of O_2_ to OH^−^ predominantly via the transfer of 4 electrons. The impressive improvement in selectivity is attributed to a binder‐induced decrease in the oxidation state of Mn, as observed during XAS investigations, resulting in a more favorable Mn^2+^:Mn^3+^:Mn^4+^ ratio, and confirming that Mn plays a major role in the ORR performance of the trimetallic catalyst. Control experiments on MnFeNiOx and commercial Mn oxides indicated, on the one hand, that the valence changes observed are neither related to the acidic nature of Nafion, nor to disproportionation, and on the other hand, that Mn^4+^ species are susceptible to chemical reduction in the presence of Nafion. Although further studies are still required to fully reveal the extent of the chemical changes, as well as the variety of materials that may be susceptible to them, it is clear that Nafion cannot always be regarded as inert, and that awareness on its use is required for investigations where the intrinsic activity of a catalytic material is the main focus of the work.

## Conflict of interest

The authors declare no conflict of interest.

## Supporting information

As a service to our authors and readers, this journal provides supporting information supplied by the authors. Such materials are peer reviewed and may be re‐organized for online delivery, but are not copy‐edited or typeset. Technical support issues arising from supporting information (other than missing files) should be addressed to the authors.

Supporting Information
