# Supplementary material for: Nafion‐Induced Reduction of Manganese and its Impact on the Electrocatalytic Properties of a Highly Active MnFeNi Oxide for Bifunctional Oxygen Conversion
Source: ChemElectroChem. 2021 Aug 16;8(15):2979–83. doi: 10.1002/celc.202100744 (PMC8457226; doi:10.1002/celc.202100744)
Supplement: Supplementary file 1 — Supporting Information [file CELC-8-2979-s001.pdf]

ChemElectroChem

Supporting Information

Nafion-Induced Reduction of Manganese and its Impact on the Electrocatalytic Properties of a Highly Active MnFeNi Oxide for Bifunctional Oxygen Conversion**

Dulce M. Morales,* Javier Villalobos, Mariya A. Kazakova, Jie Xiao, and Marcel Risch*

Supporting Information

Nafion-induced reduction of manganese and its impact on the electrocatalytic properties of a highly active MnFeNi oxide for bifunctional oxygen conversion

Dulce M. Morales,*[a] Javier Villalobos,[a] Mariya A. Kazakova,[b] Jie Xiao,[c] and Marcel Risch*[a]

[a] Nachwuchsgruppe Gestaltung des Sauerstoffentwicklungsmechanismus, Helmholtz-Zentrum Berlin für Materialien und Energie GmbH, Hahn-Meitner-Platz 1, 14109 Berlin, Germany

[b] Boreskov Institute of Catalysis, SB RAS, Lavrentieva 5, 630090 Novosibirsk, Russia

[c] Department of Highly Sensitive X-ray Spectroscopy, Helmholtz-Zentrum Berlin für Materialien und Energie GmbH, Albert-Einstein-Straße 15, 12489 Berlin, Germany

***Corresponding author** e-mail:

dulce.morales_hernandez@helmholtz-berlin.de (Dulce M. Morales)

marcel.risch@helmholtz-berlin.de (Marcel Risch)

Experimental

Chemicals

Powdered $MnSO_4$, Mn_2O_3, MnO_2, $LiNiO_2$ and FeO, as well as Nafion solution (5 vol%) were purchased from Sigma-Aldrich. NiO, and Fe_2O_3 were purchased from Roth and Alfa Aesar, respectively. These chemicals were used as received without further purification.

Synthesis of MnFeNiOx catalyst was reported previously.[1] Firstly, growth of multi-walled carbon nanotubes (MWCNTs) was carried out by chemical vapor deposition of ethylene at 680 °C using Fe_2Co/Al_2O_3 as catalyst, with subsequent acidic treatment in HCl for 4 h to remove catalyst residues. After washing to neutral pH and drying, oxygen functionalities were introduced to the obtained powder by boiling it in concentrated HNO_3 for 2 h, followed by washing with distilled water and drying, thus obtaining oxidized MWCNTs (MWCNTs-Ox). A solution containing a mixture of Mn(II), Ni(II) and Fe(III) nitrates (Sigma-Aldrich) with metal ratio of 10:7:3 was used for preparation of MnFeNiOx/MWCNTs-Ox catalyst via incipient wetness impregnation. The obtained material was dried at 110 °C for 4 h, and subsequently annealed at 350 °C for 4 h in inert atmosphere, forming thus trimetallic oxide nanoparticles with a total metal loading of 14.4 wt%, and individual metal loadings of 7.3, 5.0, and 2.1 wt% corresponding to Mn, Ni, and Fe, respectively, according to XRF studies.[1]

Elemental analysis

MnFeNiOx was examined by TEM using a Themis-Z 3.1 instrument (TFS, USA) equipped with X-FEG-monochromator and CS/S double corrector, accelerating voltage of 200 kV. Elemental analysis was performed with Super-X EDX detector (energy resolution about 120 eV) in HAADF STEM mode. The samples were prepared by ultrasonic dispersion in ethanol and subsequent deposition of the suspension upon a "holey" carbon film supported on a copper grid.

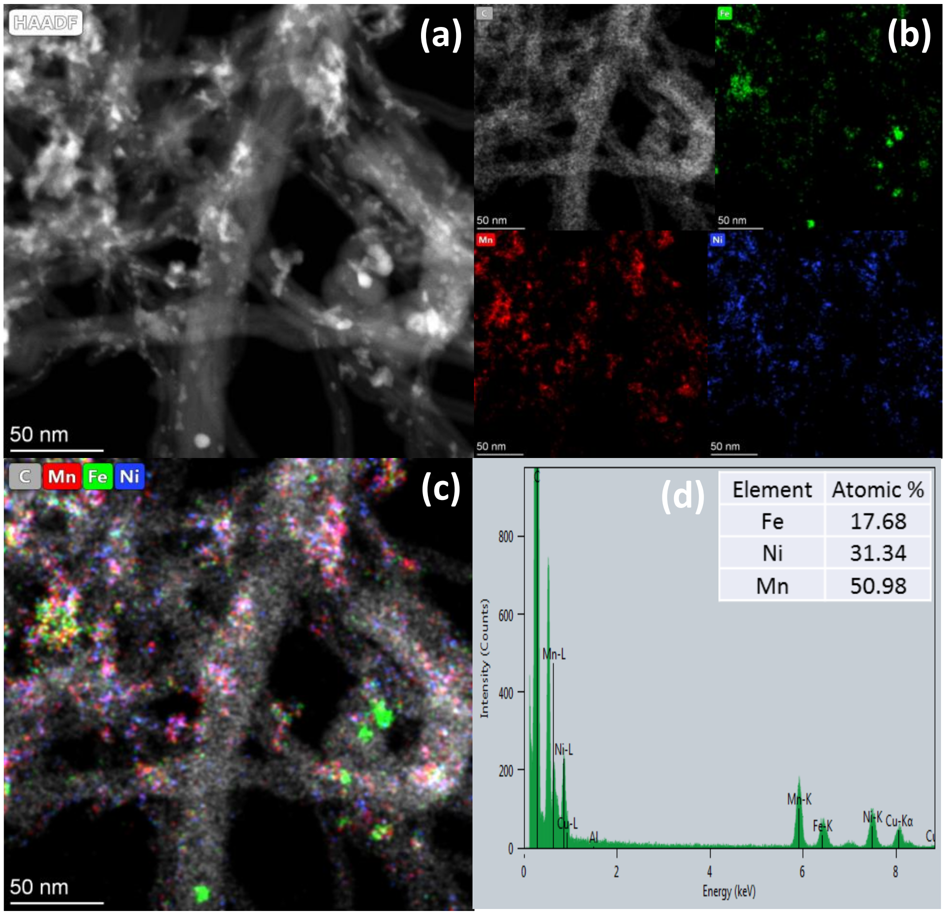

Figure S1. (a) HAADF-STEM image MnFeNiOx, (b) EDX elemental maps corresponding to C (grey), Fe (green), Mn (red) and Ni (blue), (c) combined EDX elemental map, and (d) integral EDX spectrum showing the metal composition averaged to 50 nm as an inset.

Electrochemical methods

Electrochemical measurements were conducted following the procedure illustrated in **Scheme S1**, and described in detail in this section.

Electrochemical investigations were conducted in a three-electrode configuration, single-compartment electrochemical cell. The setup was kept into a climate chamber (Binder) set to 25 °C during the measurements to ensure temperature control. The working, counter and reference electrodes were a catalyst-coated glassy carbon (GC) rotating disk electrode (4 mm diameter), a graphite rod, and a Hg|HgO|NaOH (1 M) electrode (ALS Inc.), respectively. Catalyst deposition onto the GC electrode was done by drop-casting 5.3 μL catalyst ink. The ink consisted

of 5 mg mL^{-1} of the active material dispersed in a mixture of water and ethanol (1:1 volume ratio), in the presence or absence of 2 vol% Nafion solution (~5% Nafion in a mixture of alcohols), by sonication for 15 min. The total catalyst loading on the GC electrode was 210 µg cm^{-2}. The electrolyte was 0.1 M NaOH standard solution (Sigma-Aldrich) thoroughly purged with oxygen or argon. A flowing stream of the corresponding gas was kept onto the electrolyte surface during the measurements to maintain the gas saturation. All measurements were conducted using a Reference 600+ potentiostat (Gamry) equipped with an RRDE-3A rotator (ALS Inc.).

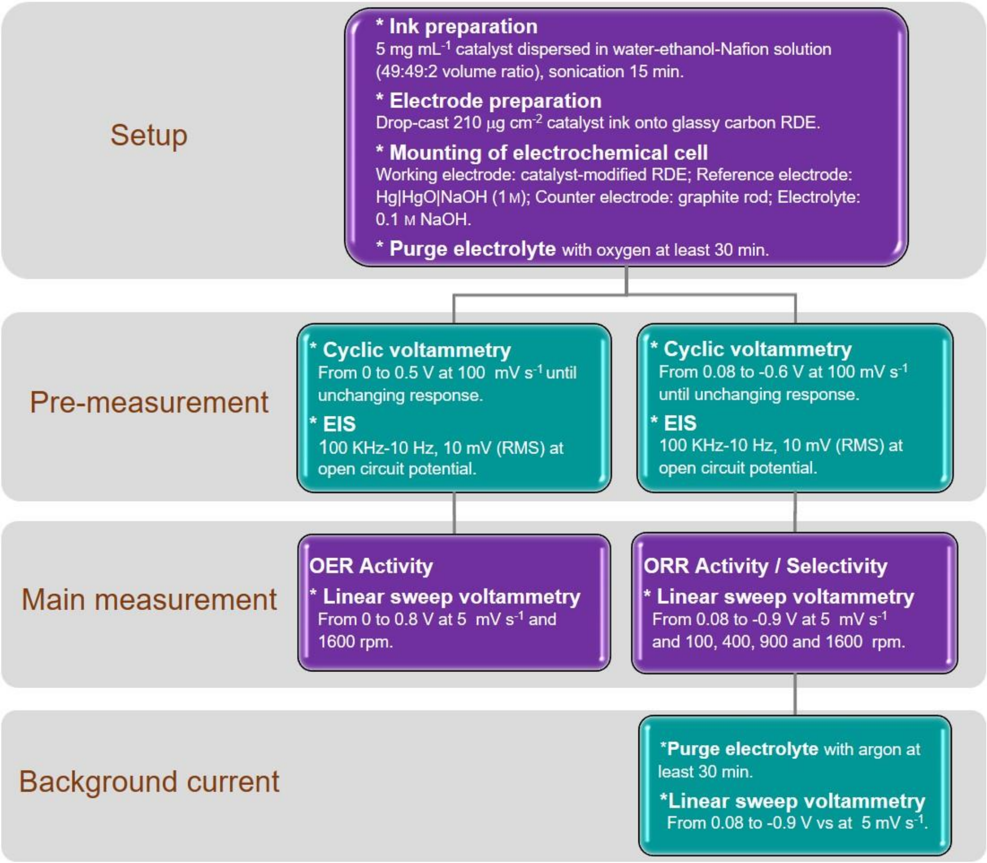

Scheme S1. Electrochemical protocol used for the investigation of catalytic properties of MnFeNiOx. Potentials are indicated with respect to the reference electrode.

Prior to the measurements, catalyst-modified electrodes were subjected to continuous potential cycling at a scan rate of 100 mV s^{-1} in the potential range from 0.08 to -0.6 V vs Hg|HgO|NaOH (prior to ORR) or from 0 to 0.5 V vs Hg|HgO|NaOH (prior to OER) until an unchanging voltammetric response was observed. Electrochemical impedance spectra were subsequently recorded in the frequency range from 100 kHz to 1 Hz with an AC amplitude of 10 mV (RMS). The uncompensated resistance (R_U) was determined from the resulting Nyquist plots, and later used to iR_U-drop-correct the measured potentials according to **Equation 1**, where i is the measured current. The obtained R_U values were in average 47 ± 4 Ω.

$$E_{corrected} = E_{measured} - i\,R_U \qquad (1)$$

Linear sweep voltammograms were afterwards recorded in the potential ranges from 0.08 to -0.9 V vs Hg|HgO|NaOH and from 0 to 0.8 V vs Hg|HgO|NaOH, for the ORR and for the OER, respectively, at a scan rate of 5 mV s^{-1} and rotation rate of 1600 rpm to evaluate the catalytic activity towards the corresponding reaction. An additional voltammogram was recorded in Ar-purged electrolyte at the same scan rate to determine the background current. All measurements were done at least in triplicate. The individual background-corrected measurements used for obtaining the average voltammograms depicted in **Figure 1a** in the main manuscript are shown in **Figure S2**.

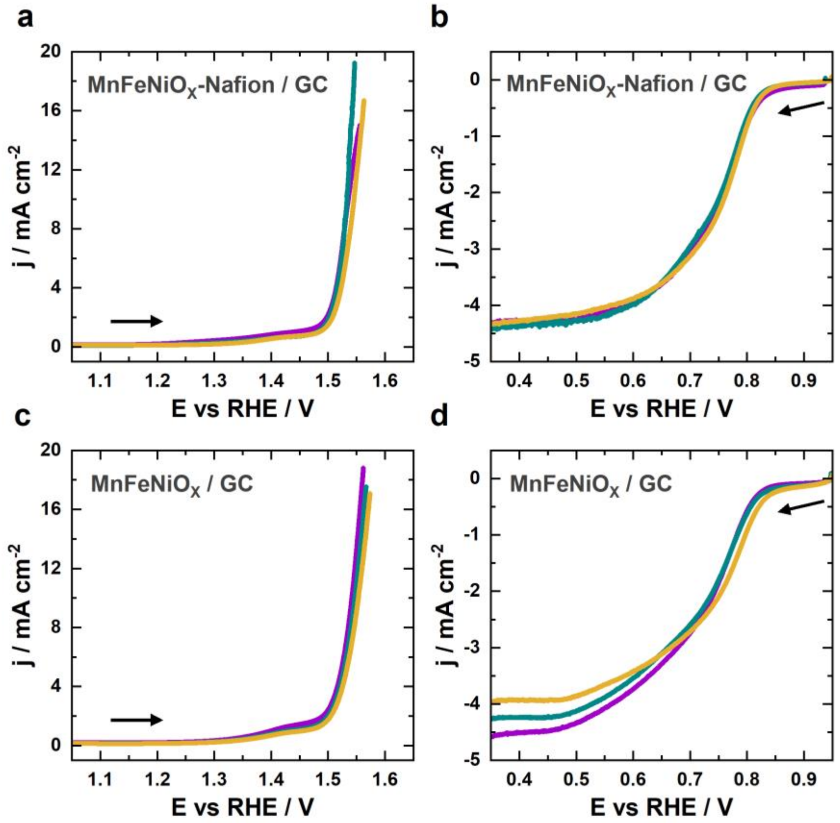

Figure S2. Linear sweep voltammograms of MnFeNiOx recorded in triplicate in O_2-saturated 0.1 M NaOH solution at a scan rate of 5 mV s^{-1} and electrode rotation of 1600 rpm in the (a,c) OER, and (b,d) ORR potential regions. Each voltammogram was recorded with a freshly prepared electrode film (a,b) in the presence and (c,d) in the absence of Nafion. Black arrows indicate the direction of the voltammetric scan.

All potentials are reported with respect to the reversible hydrogen electrode (RHE) scale. To convert the measured potentials to this scale, at the start of each experiment day the voltage between the reference electrode and an RHE electrode (Gaskatel) was measured for 10 min. The last value recorded was registered and added to the potentials measured. The average value obtained for different experiment days was 0.8795 ± 0.0216 V. Activity metrics E_{OER} and E_{ORR}, corresponding to the potentials vs RHE at which current densities of +10 and -1 mA cm^{-2} were attained, respectively, were determined from the iR_U-compensated voltammograms, and the obtained values are shown in **Table S1**. These activity metrics were chosen according to reported guidelines.[2,3]

Table S1. Activity metrics corresponding.

Sample	Activity metric	E vs RHE / V set 1	E vs RHE / V set 2	E vs RHE / V set 3	E vs RHE / V average
MnFeNiOx-Nafion/GC	E_{OER}[a]	1.535	1.533	1.546	1.538 ± 0.007
	E_{ORR}[b]	0.789	0.785	0.791	0.788 ± 0.003
MnFeNiOx/GC	E_{OER}[a]	1.542	1.547	1.553	1.547 ± 0.006
	E_{ORR}[b]	0.785	0.772	0.771	0.776 ± 0.009

[a] E vs RHE at +10 mA cm^{-2}; [b] E vs RHE at -1 mA cm^{-2}.

ORR selectivity was investigated by collecting linear sweep voltammograms in the ORR potential region at rotation rates of 100, 400, 900 and 1600 rpm. The background-corrected voltammograms reported in the main manuscript represent the average of the three independent sets of measurements shown in **Figure S3**.

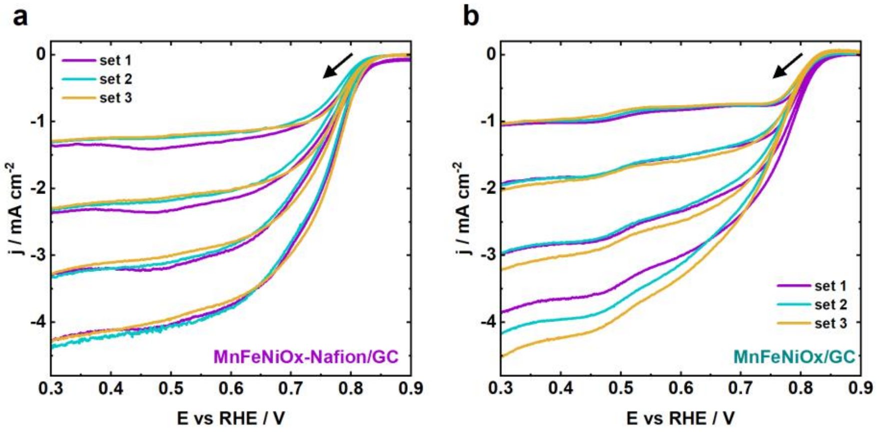

Figure S3. Comparison of three independent sets of background-corrected linear sweep voltammograms recorded at a scan rate of 5 mV s^{-1} and electrode rotation rates of 100, 400, 900 and 1600 rpm in O_2-saturated 0.1 M NaOH solution, corresponding to (a) MnFeNiOx-Nafion/GC, and (b) MnFeNiOx/GC. Black arrows indicate the direction of the voltammetric scan.

Subsequently, current density (j) was extracted from the background-corrected voltammograms at selected potentials, and the inverse of the obtained values was later plotted as a function of the square root of the angular velocity of rotation (ω), which is related to the electrode rotation rate (r) according to **Equation 2**. The linear regression obtained from the plotted data is described by the Koutecky-Levich equation (**Equation 3**):[3,4]

$$\omega = \frac{2\, r\, \pi}{60} \tag{2}$$

$$\frac{1}{j} = \frac{1}{j_k} + \frac{1}{0.62\, n\, F\, D^{2/3}\, \nu^{-1/6}\, C} \cdot \frac{1}{\omega^{1/2}} \tag{3}$$

where, j_k is the kinetic-limited current, F is the Faraday constant, D is the diffusion coefficient, ν is the kinematic viscosity, C is the bulk concentration, and n is the number of electrons transferred. With the resulting slope and considering values of D = 1.9x10^{-5} cm^2 s^{-1}, ν = 1.1x10^{-2} cm^2 s^{-1}, and

C = 1.2x10^{-6} mol cm^{-3}, corresponding to an O_2-saturated 0.1 M NaOH solution,[5] *n* was determined. **Figure S4** shows the Koutecky-Levich plots obtained with three independent measurement sets. The corresponding *n* values obtained at different potentials are summarized in **Table S2**.

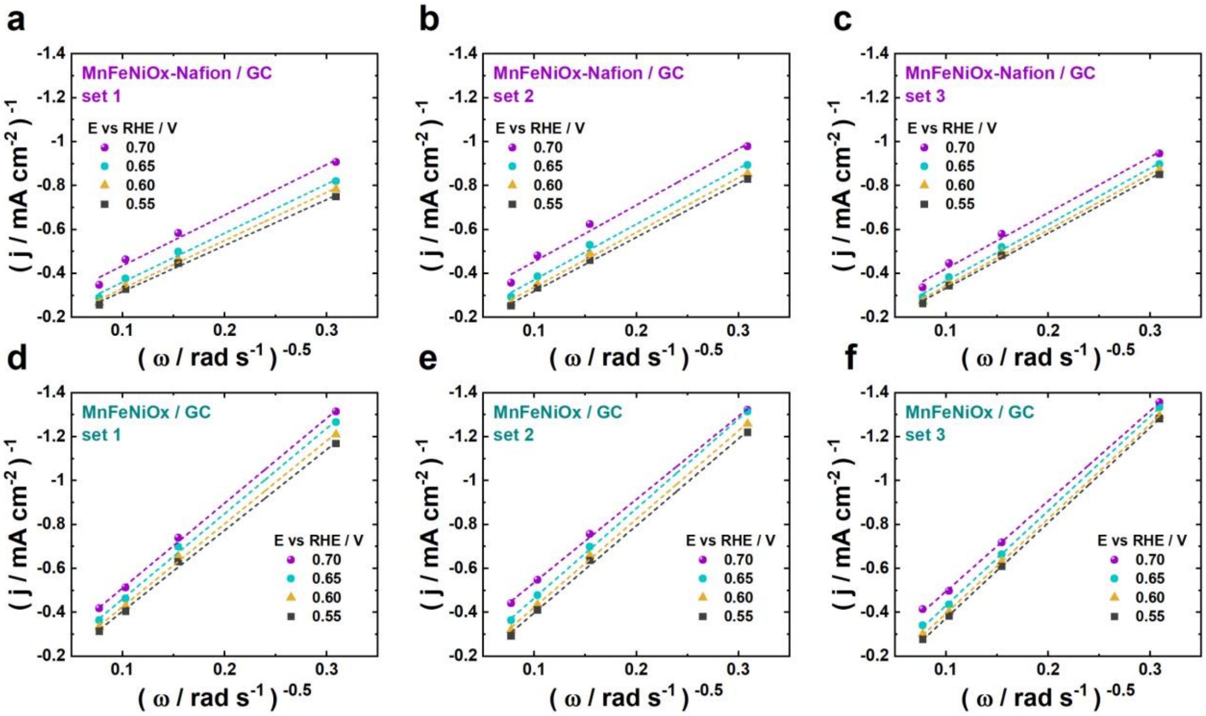

Figure S4. Koutecky-Levich plots obtained from three independent sets of linear sweep voltammograms recorded at a scan rate of 5 mV s^{-1} and electrode rotation rates of 100, 400, 900 and 1600 rpm in O_2-saturated 0.1 M NaOH solution, corresponding to (a,b,c) MnFeNiOx-Nafion/GC, and (d,e,f) MnFeNiOx/GC.

Table S2. Number of electrons transferred (n) determined from three individual sets of experiments.

Sample	E vs RHE / V	n / - set 1	n / - set 2	n / - set 3	n / - average
MnFeNiOx-Nafion/GC	0.70	4.00	3.59	3.63	3.74 ± 0.226
	0.65	4.13	3.64	3.60	3.79 ± 0.295
	0.60	4.21	3.70	3.62	3.84 ± 0.320
	0.55	4.41	3.76	3.69	3.95 ± 0.397
MnFeNiOx/GC	0.70	2.39	2.44	2.25	2.36 ± 0.098
	0.65	2.34	2.26	2.14	2.25 ± 0.101
	0.60	2.44	2.30	2.14	2.29 ± 0.150
	0.55	2.50	2.33	2.12	2.32 ± 0.190

X-ray absorption spectroscopy (XAS)

Sample preparation was done either by attaching untreated, powdered samples to carbon tape, or by dispersing the powder (5 mg mL^{-1}) in one of the following dispersion solutions by sonication for 15 min:

1. Binder-free solution (WE): a mixture of water and ethanol (1:1 volume ratio).
2. Solution containing untreated binder (Nafion): a mixture of water and ethanol (1:1 volume ratio) containing 2 vol% Nafion solution (~5% Nafion in a mixture of alcohols).
3. Solution containing ion-exchanged Nafion (Nafion-Na^+): a mixture of water and ethanol (1:1 volume ratio) with 2 vol% of a binder solution prepared according to a procedure reported elsewhere,[6] consisting of drop-wise mixing Nafion solution (~5% Nafion in a mixture of alcohols) and 0.1 M NaOH solution (2:1 volume ratio), thus exchanging H^+ for Na^+ ions.

Binder-containing dispersions were drop-cast onto glassy carbon plates or graphite foil of 5x5 mm^2 and left to dry at ambient conditions. Binder-free dispersions were poured onto a watch glass and left to dry at ambient conditions. The recovered powders were pressed onto carbon tape.

XAS measurements were carried out at the LiXEdrom experimental station at the U49/2 PGM-1 beamline at the BESSY II synchrotron (Helmholtz-Zentrum Berlin für Materialien und Energie) at room temperature.[7] The samples on carbon supports were attached to a current collector using Cu tape, and the spectra were recorded in total electron yield mode by collecting the drain current with a Keithley 6514 ammeter. Spectra were collected in the ranges from 630 to 676 eV, from 700 to 735 eV, and from 832 to 885 eV, corresponding to the Mn-L_3, Fe-L_3 and Ni-L_3 edge energy regions, respectively, at least in duplicate and in different spots on the samples to ensure reproducibility of spectra as well as to prevent radiation-induced sample damage. Calibration of energy axis and data processing were done as reported previously,[8] using the software Bessy. In short, the recorded energies were corrected by the difference in the position of the peak of maximum intensity in the Mn-L_3 edge spectrum of $MnSO_4$ with respect to 641 eV. Normalization of the spectra was conducted by dividing the recorded intensities by the photon flux, followed by subtraction of the polynomial fit (order 0 or 1) of the signal before the L_3 edge, and subsequent division by the polynomial fit (order 0 or 1) of the signals recorded after the L_2 edge. A step-by-step example can be found in the supporting information of a report by Villalobos *et al.*[8] The spectra were further normalized to a maximum intensity of 1 to facilitate their comparison.

References

[1] D. M. Morales, M. A. Kazakova, S. Dieckhöfer, A. G. Selyutin, G. V. Golubtsov, W. Schuhmann, J. Masa, *Adv. Funct. Mater.* **2020**, *30*, 1905992.

[2] a) C. C. L. McCrory, S. Jung, J. C. Peters, T. F. Jaramillo, *J. Am. Chem. Soc.* **2013**, *135*, 16977; b) W. Chen, Q. Xiang, T. Peng, C. Song, W. Shang, T. Deng, J. Wu, *iScience* **2020**, *23*, 101532.

[3] K. J. J. Mayrhofer, D. Strmcnik, B. B. Blizanac, V. R. Stamenkovic, M. Arenz, N. M. Markovic, *Electrochim. Acta* **2008**, *53*, 3181.

[4] A. J. Bard, L. R. Faulkner, *Electrochemical methods and applications*, Wiley-Interscience, New York, London, **2000**.

[5] R. Chen, H. Li, D. Chu, G. Wang, *J. Phys. Chem. C* **2009**, *113*, 20689.

[6] J. Suntivich, H. A. Gasteiger, N. Yabuuchi, Y. Shao-Horn, *J. Electrochem. Soc.* **2010**, *157*, B1263.

[7] E. F. Aziz, J. Xiao, R. Golnak, M. Tesch, *JLSRF* **2016**, *2*, A80.

[8] J. Villalobos, R. Golnak, L. Xi, G. Schuck, M. Risch, *J. Phys. Energy* **2020**, *2*, 34009.
